# Comparing models of delivery for cancer genetics services among patients receiving primary care who meet criteria for genetic evaluation in two healthcare systems: BRIDGE randomized controlled trial

**DOI:** 10.1186/s12913-021-06489-y

**Published:** 2021-06-02

**Authors:** Kimberly A. Kaphingst, Wendy Kohlmann, Rachelle Lorenz Chambers, Melody S. Goodman, Richard Bradshaw, Priscilla A. Chan, Daniel Chavez-Yenter, Sarah V. Colonna, Whitney F. Espinel, Jessica N. Everett, Amanda Gammon, Eric R. Goldberg, Javier Gonzalez, Kelsi J. Hagerty, Rachel Hess, Kelsey Kehoe, Cecilia Kessler, Kadyn E. Kimball, Shane Loomis, Tiffany R. Martinez, Rachel Monahan, Joshua D. Schiffman, Dani Temares, Katie Tobik, David W. Wetter, Devin M. Mann, Kensaku Kawamoto, Guilherme Del Fiol, Saundra S. Buys, Ophira Ginsburg, on behalf of the BRIDGE research team

**Affiliations:** 1 Huntsman Cancer Institute, 2000 Circle of Hope Drive, Salt Lake City, UT 84112 USA; 2 Department of Communication, University of Utah, 255 S. Central Campus Drive, Salt Lake City, UT 84112 USA; 3 Perlmutter Cancer Center, NYU Langone Health, 160 E. 34th Street, New York, NY 10016 USA; 4 School of Global Public Health, New York University, 726 Broadway, New York, NY 10012 USA; 5 Department of Biomedical Informatics, University of Utah, 421 Wakara Way, Suite 140, Salt Lake City, UT 84108 USA; 6 Veterans Administration Medical Center, 500 S. Foothill Boulevard, Salt Lake City, UT 84149 USA; 7 Department of Population Health, NYU Grossman School of Medicine, 550 First Avenue, New York, NY 10016 USA; 8 Department of Medicine, NYU Grossman School of Medicine, 550 First Avenue, New York, NY 10016 USA; 9 Medical Center Information Technology, NYU Langone Health, 360 Park Avenue South, New York, NY 10010 USA; 10 Department of Population Health Sciences, University of Utah, 295 Chipeta Way, Salt Lake City, UT 84108 USA; 11 NYU Langone Health, 550 First Avenue, New York, NY 10016 USA; 12 Boost Services, Epic Systems Corporation, 1979 Milky Way, Verona, WI 53593 USA; 13 Department of Pediatrics, University of Utah, 295 Chipeta Way, Salt Lake City, UT 84108 USA; 14 Department of Internal Medicine, University of Utah, 30 N 1900 E, Salt Lake City, UT 84132 USA

**Keywords:** Genetic services, Primary care, Health technology, Population health management

## Abstract

**Background:**

Advances in genetics and sequencing technologies are enabling the identification of more individuals with inherited cancer susceptibility who could benefit from tailored screening and prevention recommendations. While cancer family history information is used in primary care settings to identify unaffected patients who could benefit from a cancer genetics evaluation, this information is underutilized. System-level population health management strategies are needed to assist health care systems in identifying patients who may benefit from genetic services. In addition, because of the limited number of trained genetics specialists and increasing patient volume, the development of innovative and sustainable approaches to delivering cancer genetic services is essential.

**Methods:**

We are conducting a randomized controlled trial, entitled Broadening the Reach, Impact, and Delivery of Genetic Services (BRIDGE), to address these needs. The trial is comparing uptake of genetic counseling, uptake of genetic testing, and patient adherence to management recommendations for *automated, patient-directed* versus *enhanced standard of care* cancer genetics services delivery models. An algorithm-based system that utilizes structured cancer family history data available in the electronic health record (EHR) is used to identify unaffected patients who receive primary care at the study sites and meet current guidelines for cancer genetic testing. We are enrolling eligible patients at two healthcare systems (University of Utah Health and New York University Langone Health) through outreach to a randomly selected sample of 2780 eligible patients in the two sites, with 1:1 randomization to the genetic services delivery arms within sites. Study outcomes are assessed through genetics clinic records, EHR, and two follow-up questionnaires at 4 weeks and 12 months after last genetic counseling contactpre-test genetic counseling.

**Discussion:**

BRIDGE is being conducted in two healthcare systems with different clinical structures and patient populations. Innovative aspects of the trial include a randomized comparison of a chatbot-based genetic services delivery model to standard of care, as well as identification of at-risk individuals through a sustainable EHR-based system. The findings from the BRIDGE trial will advance the state of the science in identification of unaffected patients with inherited cancer susceptibility and delivery of genetic services to those patients.

**Trial registration:**

BRIDGE is registered as NCT03985852. The trial was registered on June 6, 2019 at clinicaltrials.gov.

**Supplementary Information:**

The online version contains supplementary material available at 10.1186/s12913-021-06489-y.

## Background

Hereditary cancer syndromes affect patients’ risks of many common, adult-onset cancers [1–5]. Pathogenic variants (i.e., variants that increase cancer risk; PVs) in cancer predisposition genes can be identified in the germline of 7% of breast, 11–15% of ovarian [2, 6–8], 10% of colorectal [3, 9, 10], 4–11% of pancreas [11], and 12% of advanced prostate cancers unselected for age, family history, or ethnicity [12, 13]. Prior evidence supports individualizing cancer screening and prevention based on cancer risk for unaffected individuals without a personal history of cancer [14–19].

Advances in genetics and sequencing technologies are allowing the identification of more individuals with an inherited cancer susceptibility who could benefit from tailored cancer screening and prevention recommendations. A growing number of genes have been associated with increased risks for breast, ovarian, colorectal, pancreas, and prostate cancers [7, 10, 11, 13, 20–24]. Use of multi-gene panel testing can more than double the rate of detection of PVs compared to testing single genes [25–28], and newer sequencing technologies are also expanding the tumor spectrum associated with particular genes. These factors are leading to a broader range of family histories being considered as an indication for genetic testing [29]. All these advances are leading to rapidly increasing numbers of unaffected patients who meet indications for genetic testing and highlight the need to develop novel ways to identify eligible patients and then deliver cancer genetics services. Our study, entitled Broadening the Reach, Impact, and Delivery of Genetic Services (BRIDGE), is addressing this gap.

### Identification of unaffected individuals with hereditary cancer syndromes

Efforts to identify individuals with inherited cancer susceptibility have generally focused on first testing patients who have had cancer with subsequent testing of their unaffected at-risk relatives once a PV is found [30–32]. However, this strategy misses families in which there is no living, affected relative to test, and may be an important barrier to accessing genetic services. Studies have begun to explore the feasibility of screening unaffected patients for inherited cancer susceptibility in primary care settings [33–36]. In these settings, cancer family history information can be used to identify those most likely to carry a PV as well as to tailor screening regardless of whether a PV is identified [37–43]. National guidelines recommend testing for unaffected individuals with a significant cancer family history [44], and the Affordable Care Act mandated coverage of genetic testing for hereditary breast/ovarian cancer for unaffected individuals with appropriate family history [45]. However, family history information is underutilized to stratify risk [46–48], leading to missed opportunities for referral to genetic testing [49]. In one study, 37% of newly diagnosed breast cancer patients found to have PVs had a family history that warranted genetic testing prior to their diagnosis [50]. The National Health Interview Survey reported that only 9.5% of unaffected women with a family history of breast and ovarian cancer had discussed genetic testing with their healthcare provider, and only 2.7% had genetic testing [51].

Novel strategies are therefore needed to facilitate the use of family history information to identify unaffected individuals who may have inherited cancer susceptibility. Although some family history information is often collected in primary care settings, barriers to collecting or using this information in clinical practice include limited visit time; competing demands; reimbursement criteria; and providers’ training, knowledge and skills [47, 52–56]. Primary care providers (PCPs) may not have specialized knowledge about genetics and genetic conditions or know who to refer for genetic services [57], and there may be limited availability of specialists outside of referral centers making the services difficult for many people to access, particularly those in rural areas. Many tools that are available to facilitate use of family history information in primary care require significant time and resources and were developed independent of workflow standards in clinics; as a result, widespread implementation of these tools has not occurred [48, 49]. System-level strategies, such as population health management, are needed to assist PCPs in identifying patients who may benefit from genetic services [58].

To address the need for a system-level population health management strategy, our team has developed an algorithm-based system to screen cancer family history information already available in the electronic health record (EHR) [59], and identify unaffected patients who receive primary care at the study sites and meet criteria for genetic evaluation for hereditary breast, ovarian, prostate, pancreas, and/or colorectal cancers [60, 61]. A pilot test conducted with 71 patients receiving primary care at University of Utah Health (UHealth) who were identified with the algorithm showed that the majority (62%) were successfully engaged through outreach from the cancer genetics team to consider genetics evaluation [62]. This algorithm forms the basis of a system-wide approach in the BRIDGE study to identify unaffected primary care patients meeting guidelines for genetic evaluation.

### Models for delivery of cancer genetic services

Once unaffected individuals at risk for a hereditary cancer syndrome are identified, genetic services (e.g., genetic counseling, genetic testing, risk-reduction recommendations) need to be delivered. The number of trained genetic specialists is limited both in capacity and location, and more sustainable, and reimbursable, service delivery models are needed to accommodate an increasing patient volume [63–65]. Therefore, the development and testing of innovative approaches to the delivery of cancer genetic services is essential.

Prior research has compared different delivery models for cancer genetic services [66]. A number of these studies have evaluated alternative modes of delivering pre- and post-test counseling by a genetic counselor [67–69]. Studies comparing in-person and telephone counseling have found telephone counseling to be acceptable to patients, with no differences in knowledge, psychosocial outcomes, satisfaction, or patient-centered communication [66, 68, 70, 71]. However, uptake of genetic testing has been shown to be lower for telephone versus in-person counseling [68], and factors such as race/ethnicity, distress, and perceived risk may impact uptake of testing [72, 73]. Other studies have reported similar levels of knowledge, satisfaction, depression, and anxiety for in-person and video counseling [66, 70, 71, 74].

Fewer studies have explored supplementing genetic counselors with other models within the healthcare system [75, 76]. As referrals for hereditary cancer risk assessment increase [66, 70, 74, 77], models that direct genetic counseling time and resources to those patients with the greatest needs for in-person genetics care are warranted. Models are also needed that can reach rural and other underserved areas that may not be near genetics providers. Patient-directed approaches, in which patients seek the genetics information and services that they need, have promise as a service delivery model. One model of genetic testing that is patient-driven and has grown greatly in popularity is direct-to-consumer (DTC) genetic testing, in which consumers can order genetic testing directly and has minimal or no involvement from a clinician or genetic counselor [78]. While DTC approaches may increase access to genetic testing, it is unknown whether DTC genetic test results will be shared with primary care providers [79] or result in appropriate clinical management [80–82]. Therefore, more effective genetic services delivery models may incorporate features of the DTC approach (e.g., patient-directed, increased access, accessible outside academic medical centers) while offering genetic testing within the healthcare system so that providers are aware of the results and have the support they need for using the results to individualize care.

Use of automated conversational agents (i.e., chatbots) may support a patient-directed genetic services delivery model for genetics education, decision making about genetic testing, and return of genetic test results. Chatbots have many advantages for patient education, including providing scripted education interactively, answering questions using natural language processing, chunking information into small segments that are easier to process, and allowing for choice in the amount of information received, consistent with adult learning recommendations [83–85]. This technology is being used in a variety of healthcare contexts, including mental health care, health promotion, and facilitation of informed decision making related to prostate cancer screening [86–90]. Chatbots are also being used in genetic services delivery such as collecting patient data, providing information, delivering results, and facilitating cascade testing of at-risk relatives [91–95]. However, user responses to the technology need to be further investigated in the context of genetics, as there is a lack of research that has compared genetic services delivery through a chatbot to standard of care genetic services delivery models using a randomized design. Research on chatbots to date indicates that users would be receptive to use of this technology in health, although concerns related to accuracy, data privacy, and lack of empathy have been raised [95, 96]. Chatbots are therefore a promising technology for use in genetic services delivery, but additional research is needed to examine their efficacy and to investigate implementation outcomes [97] within healthcare systems.

### Effects of genetic services delivery models across population subgroups

In examining the efficacy of genetic services delivery models, it is critical to explore how these effects may vary across population subgroups defined by factors such as race, ethnicity, and geographic location (i.e., rural/frontier vs. urban) to avoid worsening health disparities in access to care and health outcomes [98–100]. Prior research has shown that individuals from minority racial and ethnic groups have decreased access to and utilization of genetic services [101–104], even when cost barriers are minimized [105], and that race and ethnicity can modify the effects of genetic services delivery models. A randomized trial comparing in-person and telephone-based genetic counseling found that the effect of mode of delivery differed by race; women from minority groups who were assigned to telephone counseling were the least likely to complete genetic testing [72]. Less research has examined how geographic location may modify the effects of genetic services delivery models. Individuals in rural (< 100 persons per square mile) and frontier (< 7 persons per square mile) [106] areas in the U.S. have less access to care [107] and worse health outcomes [108–112], In rural and frontier regions, clinics and hospitals can be hundreds of miles from patients’ homes, so rural and frontier residents have less access to specialty genetics care [56, 113, 114]. In addition, clinical sites in rural areas may not have providers with specialized genetics knowledge [115]. These prior findings indicate that exploring whether genetic services delivery models have similar effects across population subgroups is critical.

### Aims of BRIDGE study

To address the research needs of developing system-wide population health management strategies to identify unaffected patients at risk for inherited cancer syndromes and delivering cancer genetic services effectively across population subgroups, we are conducting the BRIDGE randomized controlled trial (RCT) with patients who receive primary care at two large healthcare systems. The BRIDGE trial has the following specific aims:

1. Identify unaffected patients receiving primary care who qualify for genetic risk assessment in two healthcare systems (UHealth and New York University Langone Health [NYULH]) using a clinical decision support algorithm to automatically evaluate family history of cancer through a population health management approach.
2. Compare uptake of genetic counseling and testing for intervention (i.e., active outreach and automated, patient-directed education via a chatbot) vs. enhanced standard of care (i.e., active outreach followed by standard of care) genetic services delivery models among unaffected patients who receive primary care at each site.
3. Compare adherence to clinical care recommendations and patient cognitive and affective responses for the genetic services delivery models.

The primary outcomes of the RCT will be uptake of genetic counseling, uptake of genetic testing, and, among those who receive genetic services, adherence to management recommendations from genetics providers. We hypothesize that uptake of genetic counseling and genetic testing will be equivalent between the two genetic services delivery models. We will also explore how race, ethnicity, and geographic location modify the effects of the cancer genetics services delivery models on these outcomes. In addition to the trial outcomes, we will assess implementation outcomes based on the RE-AIM (Reach, Efficacy, Adoption, Implementation, and Maintenance) framework (Table 1) [116, 117].

**Table 1:** BRIDGE implementation outcomes

Dimension	Metric
Reach (number of patients reached)	• Identification of patients who may have inherited cancer susceptibility (Aim 1) • Identification of patients by race/ethnicity and rural/frontier vs. urban (i.e., representativeness) (Aim 1) • Uptake of genetic counseling and genetic testing by race/ethnicity and rural/frontier vs. urban (Aim 2)
Efficacy (impact on clinical outcomes)	• Uptake of genetic counseling and genetic testing for genetic services delivery models (Aim 2) • Adherence to cancer prevention and screening recommendations (Aim 3) • Differences in impact of model by race/ethnicity and rural/frontier vs. urban (Aims 2 and 3)
Adoption (number of trial participants)	• Proportion of patients in patient-directed arm who complete pre-test education and request genetic testing (Aim 2) • Proportion of patients in enhanced standard care arm who complete pre-test counseling and request genetic testing (Aim 2)
Implementation (fidelity to protocol)	• Patient response to patient portal messages (Aims 1 and 2) • Completion of workflow by genetic counseling assistants and genetic counselors (Aim 2)
Maintenance (extent a program becomes institutionalized)	• Use of the clinical decision support algorithms to identify patients after trial enrollment is complete • Continued use of genetic services delivery models after the trial is complete

## Methods

### Study overview

**Fig. 1:**
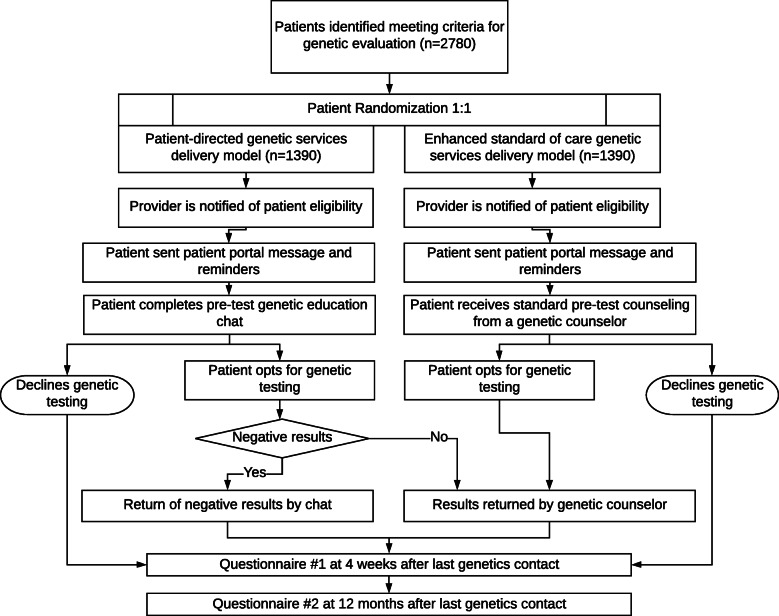
Study flow diagram for BRIDGE protocol

We are conducting a RCT to compare uptake of genetic counseling and testing and patient adherence to management recommendations for *automated, patient-directed* versus *enhanced standard of care* cancer genetics services delivery models. We are enrolling unaffected patients receiving primary care who are eligible for cancer genetic testing based on current National Comprehensive Cancer Network (NCCN) guidelines at two healthcare systems (UHealth and NYULH). We are enrolling patients through outreach to a randomly selected sample of 2780 patients (1280 at UHealth and 1500 at NYULH). Eligible patients are identified using an open clinical decision support (CDS) infrastructure designed to extract cancer family history information available in the EHR. Identified patients are randomized 1:1 at each site prior to outreach to the study arms: *automated, patient-directed* intervention genetic services delivery model and *enhanced standard of care* control genetic services delivery model (Fig. 1). The study design is based on patient-level randomization because the outreach and clinical care in both arms is delivered centrally through the genetics clinic in each system with minimal need for involvement by the primary care clinic or PCP. Study outcomes are assessed through the EHR and two follow-up questionnaires at about 4 weeks and 12 months after last genetic counseling contact. The trial has been approved as a single IRB protocol by the participating universities.

### Participants

We have created an open standards-based clinical decision support algorithm that identifies patients who receive primary care at the study sites and are eligible for genetics evaluation [62]. The algorithm utilizes structured cancer family history data available in the EHR [59] and identifies unaffected patients who meet modified 2018 NCCN guidelines for genetic testing for hereditary breast/ovarian and colorectal cancer syndromes based on their family history (Table 2). The platform was designed to be interfaced with different EHR systems via the Health Level Seven International Fast Healthcare Interoperability Resources (FHIR) standard and was deployed initially in the Epic® EHR in use at the UHealth healthcare system. UHealth is one of the largest healthcare systems in the Intermountain West region, providing care for 1.2 million residents of six states in a referral area encompassing more than 10% of the continental U. S with vast rural and frontier regions. We have adapted the algorithm for the NYULH system, which serves a large, diverse population in New York, including over 300 ambulatory sites and affiliate hospitals in Manhattan, Brooklyn, and Long Island.

**Table 2:** Adapted criteria for genetic testing to identify eligible participants

FDR or SDR diagnosed with the following regardless of age: ovarian cancer, pancreas cancer	
FDR or SDR diagnosed with the following < 50 years of age: breast cancer, colorectal cancer, endometrial cancer	
Three or more relatives on the same side of the family diagnosed with the following clusters of cancer regardless of age:	
Breast cancer, ovarian cancer, pancreas cancer, prostate cancer	
Colorectal cancer, endometrial cancer, ovarian cancer, pancreas cancer, cancer of the urinary tract, brain, small intestine	
Melanoma, pancreas cancer	
Ashkenazi Jewish ancestry and family history of breast cancer, ovarian cancer, pancreas cancer, prostate cancer	

*FDR* first-degree relative, *SDR* second-degree relative

In addition to having a cancer family history meeting NCCN criteria for genetic evaluation, all patients identified as eligible for the trial are English- or Spanish-speaking, between the ages of 25 and 60 years, had a primary care appointment in one of the two participating healthcare systems in the previous 3 years, have not had a prior cancer diagnosis other than non-melanoma skin cancer, and have not had prior genetic counseling or testing related to hereditary cancer. We selected the 25–60 years age range because screening and prevention recommendations can be modified for those in this range with inherited cancer susceptibility or familial risk [118–122]. For the study we are defining primary care as internal medicine and family medicine at both sites. In addition, because many patients (23%) in the UHealth system receive primary care through obstetrics/gynecology clinics, we will also select UHealth patients who have had a primary care appointment in these clinics in the previous 3 years. To be eligible for the trial, patients need to have an electronic patient portal (MyChart in Epic®) account because of its use for outreach in both study arms. Patients identified as meeting the algorithm who do not have an account are contacted by mail or telephone about creating one. Those who opt not to create an account are ineligible for the trial but are offered standard of care genetic services.

### Procedures

The list of patients receiving primary care who are identified by the CDS algorithm as meeting criteria for genetic evaluation was entered into a population health registry in Epic® at each of the two sites, which allows the registry to be used for tracking eligible patients and managing outreach. The registry indicates to which arm the patient is randomized, with 1:1 randomization to the patient-directed or enhanced standard of care arm. Randomization was predetermined by the study biostatistician, who has no patient contact and is masked to patient condition. One week prior to outreach in either arm, a patient’s PCP is notified that the patient will be contacted. The subsequent procedures then vary between study arms as described below.

#### Enhanced standard of care arm

In the control *enhanced standard of care* arm, patients are first sent an electronic message through the MyChart patient portal indicating that genetic services are recommended for them. The message encourages patients to contact the genetics clinic within the appropriate healthcare system (UHealth or NYULH) to schedule a genetic counseling appointment. If the patient does not respond to the message, they receive a reminder patient portal message 1 week later and up to two follow-up telephone calls from a genetic counseling assistant (GCA). Procedures after this point follow standard workflow of the clinics. Interested patients are scheduled for a usual care pre-test genetic counseling appointment. These visits may be conducted by phone, telehealth, or in person at a designated time during clinic hours and with a certified genetic counselor following clinical standard of care. Patients who choose to proceed with genetic testing can give a sample of blood or saliva if seen in person. If counseled by telephone or telehealth, they are sent saliva collection kits which can be returned by mail; this service is offered by several commercial laboratories. All genetic test results are reviewed and returned by a genetic counselor either by phone or in person, based on patient preference. A copy of the results and letter with tailored cancer screening recommendations prepared by a genetic counselor are returned to the PCP. Patients who do not respond to initial outreach attempts are sent a final electronic message letting them know how to access genetic services in the future.

#### Automated, patient-directed arm

In the intervention *automated, patient-directed* arm, patients are first sent an electronic message through the MyChart patient portal indicating that genetic services are recommended for them. The message includes a link with instructions to complete the pre-test education chat with the chatbot through the online platform. If the patient does not complete the chat, they receive a reminder patient portal message 1 week later and up to two follow-up telephone calls from a GCA. Following the completion of the pre-test education chat, a transcript is added into the patient’s EHR as an outreach encounter to document the education received. A GCA contacts each patient who completes the chat to review their decision about testing and complete a comprehensive family history. If the patient opts to proceed with testing, the GCA explains the genetic testing process and payment options and places the genetic testing order. The encounter is documented in the EHR using a clinic note template that is signed by a genetic counselor.

Patients in this arm who opt to have genetic testing are sent a saliva collection kit as described above. If the genetic sample is not returned to the lab within 30 days of order, the patient receives up to one reminder phone call and two electronic messages through the patient portal. All genetic test results are reviewed by a genetic counselor. Patients with negative results receive an electronic message in the patient portal which contains a link to a negative return of results chat with the chatbot. Transcripts of completed negative return of results chats are added to the patient’s EHR to document the encounter. Patients with PVs or variants of uncertain significance (VUS) have results returned by a genetic counselor via a telephone or telehealth visit. All patient test results (i.e., positive, VUS, negative) are uploaded to the patient portal along with a letter of tailored screening recommendations written by the genetic counselor. The patient’s PCP is also sent a copy of all documentation via Epic.

Both chats in the automated, patient-directed arm (i.e., pre-test genetic education and negative return of results) utilize the Invitae technical platform and chatbot interface and were scripted for the BRIDGE study by an interdisciplinary team of genetic counselors, genetic counseling assistants, communication scientists, and research staff. The chat content was developed through an iterative process of review of recorded genetic counseling encounters and feedback by certified genetic counselors in order to most closely represent key content delivered during in-person genetic counseling appointments. The pre-test education chat is introduced by a short video from a lead genetic counselor at each site and then provides educational content about heritability of cancer, genetic testing process, multi-gene panel tests, and possible genetic testing outcomes. The negative return of result chat informs the patient of their negative results and educates the patient about what these results mean for their long-term care. For both chats, patients move through a core set of information and then may request additional information on topics they choose. Both chats also provide the patient with the opportunity to ask questions in a free-text format. The platform utilizes a question bank so that the chatbot can respond automatically to most inquiries with a response pre-scripted by the research team, using natural language processing to determine the most appropriate response. If the system cannot match an answer to the patient’s question, the platform forwards the question to the GCA for follow up.

Patients from both arms who decide not to test receive a letter summarizing their risks and recommendations based on the available personal and family history information. In addition, patients from both arms are offered the option to schedule follow-up appointments in the genetics clinics, and these contacts and appointments are tracked.

#### Genetic testing

All genetic testing is performed at a Clinical Laboratory Improvement Act (CLIA)-certified commercial laboratory and, for NYULH patients, New York State approved laboratory. Patients receive a pan-cancer, multigene panel test for cancer susceptibility genes that includes approximately 34–36 genes. As in clinical practice, the choice of laboratory and specific multigene panel test is based on the patient’s need, services provided, and the patient’s insurance/financial status. Testing is billed to insurance, or patients can opt to self pay. Patients who are uninsured or lack coverage for genetic testing can also be assisted in applying for aid through programs offered by the laboratories. The out-of-pocket cost of genetic testing is typically between $0–$250 based on prior clinical experience, with most paying less than $100.

### Measures

In order to collect data for the trial and implementation outcomes for participants who receive genetic counseling, we use a combination of questionnaires and abstraction of EHR data.

#### Questionnaires

Patients who complete pre-test counseling in either arm are asked to complete two follow-up questionnaires, with different versions for those who did and did not opt for genetic testing. The first follow-up questionnaire is administered about 4 weeks after the last genetic counseling contact and the second is administered 12 months after the last contact. As shown in Table 3, the 4-week follow-up questionnaire assesses perceptions of genetic services, decision regret, cancer risk perceptions, genetic knowledge, numeracy, and sociodemographic characteristics. For those who have testing, we also assess cognitive (i.e., recall, comprehension), affective (i.e., test-related distress, uncertainty, positive reactions), and communication (i.e., family, provider) responses. The 12-month follow-up questionnaire assesses cancer screening and prevention behaviors, genetic discrimination, eHealth literacy, genetic self-efficacy, and sources of health information. For those who have testing, we also assess retention, risk perceptions, and communication about test results at 12 months. Patients may complete follow-up questionnaires online, by telephone, or by mail. Online questionnaires are administered via REDCap, which has a HIPAA-compliant interface. For any patients who would like to complete the questionnaires by telephone, a research coordinator masked to their individual test results administers the questionnaire. Patients receive a $10 electronic gift card as an incentive for each questionnaire. The sources of the validated items used in the questionnaires are indicated in Table 3. The questionnaires were tested as part of a pilot study at the University of Utah prior to the launch of the RCT. Questionnaires can be found as Supplemental materials.

**Table 3:** Items included in 4-week and 12-month follow-up questionnaires

Construct	Item description	Asked only of those completing testing	Assessment point(s)	
			4-week	12-month
**Perceptions of genetic services**				
Satisfaction with genetic counseling	6 items to assess participant satisfaction with the process and content of genetic counseling [123]		X	
Perceptions of genetic counseling	4 items assessing perceptions of genetic counseling and interactions with genetic counselor [124]		X	
Perceptions of clarity and helpfulness of information	2 items assessing perceptions of understandability and helpfulness of information received during genetic counseling [125] and 2 items on ability to ask questions		X	
Financial aspects of genetic testing	3 items assessing perceptions of affordability of genetic testing and insurance and billing concerns		X	
**Cognitive responses**				
Recall and interpretation	6 items assessing whether participants recalled their genetic test result and how they interpreted that result in terms of cancer risks [126]	X	X	X
Perceptions of genetic test results	3 items assessing perceptions of clarity, time spent thinking about results, and surprise with results [125]	X	X	X
Perceived utility	7 item measure assessing perceived utility of genetic test results [127]	X	X	
Decision regret	5 item measure assessing regret related to decision to receive genetic testing or not [128]		X	
Cancer risk perceptions	6 items assessing absolute and relative risk perceptions for breast, colon, and (if female) ovarian cancer		X	X
Genetic knowledge	16 item measure assessing genetic knowledge related to multigene panel testing [129]		X	
**Emotional responses**				
Distress, uncertainty, and positive experiences	21 item measure with three subscales (i.e., distress, uncertainty, positive experiences) assessing multidimensional impact of genetic test results [130]	X	X	
**Behavioral responses**				
Cancer screening	14 items assessing use of different cancer screening tests in past year			X
Surgical decisions	1 item assessing surgical procedures to reduce risk of cancer			X
Cancer prevention	11 items assessing use of cancer prevention approaches in past year			X
**Communication**				
Communication with health care providers	2 items assessing whether patient has discussed genetic test results with primary care or other health care provider [125]	X	X	X
Communication with family members	2 items assessing whether patient has discussed genetic test results with family members [125]	X	X	X
**Patient characteristics**				
Sociodemographic characteristics	Assessing educational attainment, race, ethnicity, marital status, household income, zip code, health insurance status, and having a primary care provider		X	
Diagnosis with cancer	1 item assessing diagnosis with cancer in past year			X
Genetic testing	1 item assessing use of other genetic testing in past year			X
Genetic discrimination	1 item assessing experiences of discrimination following genetic testing	X		X
Numeracy	6 item measure assessing subjective numeracy ability and preferences [131]		X	
Health literacy	3 items assessing patients’ subjective health literacy [132]			X
eHealth literacy	8 item measure assessing patient’s eHealth literacy [133]			X
Genetic self-efficacy	3 item measure assessing patients’ confidence in their ability to assess and discuss genetic risk information [134]			X
Health information seeking	5 items assessing health information seeking through different media and interpersonal channels [135, 136]			X
Health information orientation	13 items assessing patients’ perceived importance of health [137]			X
Impacts of coronavirus	10 items assessing the financial, health, and psychological impact of or diagnosis with the coronavirus (COVID-19) [138]		X	X

#### EHR data

Queries of structured data elements are used to abstract data from the EHR related to patients’ cancer family history and adherence to clinical management recommendations (e.g., breast MRI) at 12 months following genetic services. Data will be abstracted from the population health management registries at each site related to uptake of genetic counseling, uptake of genetic testing, and genetic test results.

### Analysis

For the primary trial outcome analyses, we will first check randomization by comparing demographic characteristics between patients in the two arms using bivariate statistics (t-test, Wilcoxon Ranked-Sum test, chi-squared test, or Fisher’s exact test). We will examine descriptive statistics, and will use chi-squared tests (or the nonparametric Fisher’s Exact test) with a two-sided 0.05 significance level for univariate comparison of proportions to examine the association between study arm and the primary outcomes (i.e., uptake of genetic counseling, uptake of genetic testing, adherence to clinical recommendations). We will next build multilevel multivariable logistic regression models to examine the effect of study arm on the outcome controlling for clinic-level (clinic type, patient volume, patient composition, number of providers), provider-level (specialty, years in practice, gender), and patient-level (i.e., sociodemographic, clinical) variables. Although the study design utilizes patient-level randomization and genetic counseling services are delivered centrally at both study sites, we will examine the intra-class correlation (ICC) by clinic and provider. If a significant ICC is observed, the regression models will be extended to include random effects for clinic or provider, and fit using software for generalized mixed models. Covariates will be included in models if significant in unadjusted analyses or if significantly different between arms. Using the multivariable regression models with interaction terms, we will explore whether the effects of study arm on uptake of genetic testing is modified by race/ethnicity or rurality. We will conduct both site-specific analyses and overall trial analyses. Statistical significance will be assessed as *p* < 0.05; SAS 9.4 and/or Stata 16.1 will be used for analysis.

### Power

Sample size (outreach to 1280 patients at UHealth and 1500 at NYULH) is based on pilot tests of both arms at the two study sites in order to enroll approximately the same number of patients at each site. With outreach to 640 patients per arm at UHealth and 750 at NYULH, we will achieve 80% power to detect equivalence for the uptake of genetic counseling outcome in site-specific analysis. For this analysis, the margin of equivalence, given in terms of the difference, extends from − 0.08 to 0.17. The actual difference is 0.11, and the reference group proportion is 0.18. This sample size will be sufficient to examine the uptake of genetic testing outcome as well. For that outcome, 523 patients per arm at each site achieves 80% power to detect equivalence. The margin of equivalence, given in terms of the difference, extends from − 0.04 to 0.11. The actual difference is 0.05, and the reference group proportion is 0.14.

## Discussion

The BRIDGE trial is designed to address the need to develop system-wide population health management strategies to deliver cancer genetic services to unaffected primary care patients. The primary outcomes of the trial will be uptake of genetic counseling, uptake of genetic testing, and, among those who receive genetic services, adherence to management recommendations from genetics providers. We hypothesize that uptake of genetic counseling and genetic testing will be equivalent for the automated, patient-directed genetic services delivery model delivered by a chatbot compared with the enhanced standard of care models. We will also explore whether race, ethnicity, and rurality modify the effects of the cancer genetics services delivery models on these outcomes.

BRIDGE is addressing critical clinical needs of increasing hereditary cancer testing in appropriate populations and identifying individuals with inherited cancer susceptibility [139]. Innovative aspects of the trial include testing delivery of cancer genetic services via an automated chatbot for pre-test genetics education and return of negative genetic test results. While chatbots have been used previously in delivery of some genetic services, outcomes from this modality have not been compared with standard of care in a randomized trial. Utilization of chatbots to deliver some components of genetic services has a strong potential to increase volume and access to these services for patients receiving primary care. The patient population of unaffected primary care patients identified through an EHR-based algorithm is also novel, and tests a sustainable system-level population health management approach to identification and delivery of cancer genetics services. BRIDGE also has the advantage of being conducted at two sites (UHealth and NYULH) located in different regions of the country, which have clinical structures and patient populations that are unique from one another. This allows for the examination of multiple outcomes between two unique healthcare systems. The findings from the BRIDGE trial will therefore advance the state of the science in identification of unaffected patients at increased risk for cancer and delivery of genetic services to those patients.

## Supplementary Information

**Additional file 1.**

## Data Availability

Not applicable. This protocol paper does not contain any data.

## Abbreviations

BRIDGE: Broadening the Reach, Impact, and Delivery of Genetic Services
CDS: Clinical decision support
CLIA: Clinical Laboratory Improvement Act
DTC: Direct-to-consumer
EHR: Electronic health record
FHIR: Health Level Seven International Fast Healthcare Interoperability Resources
GCA: Genetic counseling assistant
ICC: Intra-class correlation
NCCN: National Comprehensive Cancer Network
NYULH: New York University Langone Health
PCP: Primary care provider
RCT: Randomized controlled trial
RE-AIM: Reach, Efficacy, Adoption, Implementation, and Maintenance
UHealth: University of Utah Health
VUS: Variant of uncertain significance
