# Supplementary material for: Comparing models of delivery for cancer genetics services among patients receiving primary care who meet criteria for genetic evaluation in two healthcare systems: BRIDGE randomized controlled trial
Source: BMC Health Serv Res. 2021 Jun 2;21:542. doi: 10.1186/s12913-021-06489-y (PMC8170651; doi:10.1186/s12913-021-06489-y)
Supplement: Supplementary file 1 — Additional file 1. [file 12913_2021_6489_MOESM1_ESM.zip › 12913_2021_6489_MOESM1_ESM/Supplemental file Questionnaire 1 _ESM.pdf]

Questionnaire #1– Participants who receive genetic testing

Our team is doing a study called BRIDGE to better understand how to deliver genetics care to patients and we are interested in hearing your thoughts. You will find additional information about the study attached to the invitation email. If you would like to participate in the study, please complete this questionnaire.

Section 1:

For our first set of questions, we would like to ask about your experience making a decision to receive genetic testing. For each of the following questions please think about the [chat with the genetics information assistant, GIA/appointment with a genetic counselor] and mark the best answer for you.

	Strongly disagree	Somewhat disagree	Neither agree nor disagree	Somewhat agree	Strongly agree
1. [GIA/My genetic counselor] helped me to identify what I needed to know to make decisions about what would happen to me.	O	O	O	O	O
2. I felt better informed about my health after [the chat with GIA/ meeting with my genetic counselor].	O	O	O	O	O
3. The [chat with GIA/ genetic counseling session] was about the right length of time I needed.	O	O	O	O	O
4. [GIA/My genetic counselor] was truly concerned about my well-being.	O	O	O	O	O
5. The [chat with GIA/ genetic counseling session] was valuable to me.	O	O	O	O	O
6. [GIA/ My genetic counselor] seemed to understand the stresses I was facing.	O	O	O	O	O

7. Overall, how satisfied have you been with the education you have received from [GIA/your genetic counselor]?
   O Not at all satisfied
   O A little bit satisfied
   O Somewhat satisfied
   O Very satisfied

8. How convenient have you found the [education/genetic counseling] process?
   O Not at all convenient
   O A little bit convenient
   O Somewhat convenient
   O Very convenient

9. How easy or difficult was it for you to maintain your attention during the [education/genetic counseling] process?
   O Very difficult
   O Somewhat difficult
   O Somewhat easy
   O Very easy

10. How well was [GIA/your genetic counselor] able to provide you with emotional support?
   O Not at all
   O A little bit
   O Somewhat
   O Very much

11. On a scale from 1 to 5, with 1 meaning "very difficult to understand" and 5 meaning "very easy to understand," how would you rate the information you received during [the chat with GIA/ genetic counseling]?

Very difficult to understand	Somewhat difficult to understand	Neither easy nor difficult to understand	Somewhat easy to understand	Very easy to understand
1	2	3	4	5

12. On a scale from 1 to 5, with 1 meaning "not at all helpful" and 5 meaning "very helpful," how helpful was the information you received during [the chat with GIA/ genetic counseling]?

Not at all helpful		Somewhat helpful		Very helpful
1	2	3	4	5

13. After [your interaction with GIA/appointment with a genetic counselor], did you have questions for the genetic counseling team?
   O Yes
   O No
   O Don't know

If yes, on a scale from 1 to 5, with 1 meaning "very difficult" and 5 meaning "very easy," how easy or difficult was it to get the answers to these questions?

Very difficult	Somewhat difficult	Neither easy nor difficult	Somewhat easy	Very easy
1	2	3	4	5

14. Is there anything else you would like to tell us about [your chat with GIA/appointment with a genetic counselor]?

____

Section 2:

For the next set of questions, we would like you to think about the genetic test results that you received.

1. How did you receive your genetic test results?
   - O From a genetic counselor by phone
   - O From a genetic counselor in person
   - O By mail
   - O Through MyChart

2. What was your genetic test result?
   - O Positive
     - ***If positive, what would you say is the meaning of the positive result?***
       - ▪ A genetic mutation was identified and, therefore, I will develop cancer.
       - ▪ A genetic mutation was found and, therefore, I have an increased chance of developing cancer.
   - O Negative
     - ***If negative, what would you say is the meaning of the negative result?***
       - ▪ No genetic mutation was identified and, therefore, I will not develop cancer.
       - ▪ No genetic mutation was found and, therefore, I am less likely to develop cancer.
       - ▪ No genetic mutation was identified, and these results give me no information about my risk for developing cancer.
   - O VUS
     - ***If VUS, what would you say is the meaning of the VUS result?***
       - ▪ A genetic change was found but it is not certain what effect this has on my chance of developing cancer.
       - ▪ A genetic change was found that means that I have an increased chance of developing cancer.
       - ▪ A genetic change was found that means that I have a decreased chance of developing cancer.
   - O My test results are not clear to me.

3. Did your genetic test results indicate that you were at increased risk of:

Breast cancer?	YES	NO	DON'T KNOW
Ovarian cancer? (If female)	YES	NO	DON'T KNOW
Colon cancer?	YES	NO	DON'T KNOW
Any other cancer?	YES	NO	DON'T KNOW

If yes to any other cancer, which cancer(s)? Select all that apply.

a. Choose an item.

4. On a scale from 1 to 5, where 1 means “not at all” and 5 means “all of the time,” how often would you say you have thought about your test results since you received them?

Not at all		Some of the time		All of the time
1	2	3	4	5

5. On a scale from 1 to 5, where 1 means “not at all clear” and 5 means “very clear,” how clear did you think your results were?

Not at all clear		Somewhat clear		Very clear
1	2	3	4	5

6. Were you surprised by your genetic results?

O YES   O NO   O DON’T KNOW

If yes, what surprised you? ____

Please think about whether you agree or disagree with each of the following statements about your test results and mark the best box.

	Strongly disagree	Somewhat disagree	Neither agree nor disagree	Somewhat agree	Strongly agree
7. I am confident in the quality and accuracy of my results.	O	O	O	O	O
8. The educational materials provided about my results were adequate.	O	O	O	O	O
9. Having genetic testing made me feel like I have more control over my health.	O	O	O	O	O
10. I am disappointed that my results did not tell me more information.	O	O	O	O	O
11. The information I received has influenced how I will manage my health in the future.	O	O	O	O	O
12. What I learned can help reduce my chances of getting cancer.	O	O	O	O	O
13. I learned something to improve my health that I didn’t know before.	O	O	O	O	O

Section 3:

The questions below are about some specific responses you may have had after receiving your test results. Please indicate whether you have experienced each statement never, rarely, sometimes, or often in the past week.

	Never	Rarely	Sometimes	Often
1. Worrying about my risk of getting cancer	O	O	O	O
2. Being uncertain about what my test results mean about my cancer risk.	O	O	O	O
3. Being uncertain about what my test results mean for my child(ren) and/or family's cancer risk.	O	O	O	O
4. Having difficulty making decisions about cancer screening or prevention (e.g., having preventive surgery or getting medical tests done).	O	O	O	O
5. Feeling frustrated that there are no definite cancer prevention guidelines for me.	O	O	O	O
6. Thinking about how my test results have affected my work or family life.	O	O	O	O
7. Understanding clearly my choices for cancer prevention or early detection.	O	O	O	O
8. Feeling concerned about how my test results will affect my insurance status	O	O	O	O
9. Having difficulty talking about my test results with family members.	O	O	O	O
10. Worrying that the genetic counseling and testing process has brought about conflict within my family.	O	O	O	O
11. Feeling upset about my test results	O	O	O	O

	Never	Rarely	Sometimes	Often
12. Feeling sad about my test results	O	O	O	O
13. Feeling anxious or nervous about my test results	O	O	O	O
14. Feeling guilty about my test results	O	O	O	O
15. Feeling a loss of control	O	O	O	O
16. Having problems enjoying life because of my test results	O	O	O	O
17. Feeling relieved about my test results	O	O	O	O
18. Feeling happy about my test results	O	O	O	O
19. Feeling regret about getting my test results	O	O	O	O
20. Feeling that my family has been supportive during the genetic counseling and testing process	O	O	O	O
21. Feeling satisfied with family communication about my genetic test results	O	O	O	O

Section 4:

Next, reflect on your decision whether or not to receive genetic testing. Please show how strongly you agree or disagree with these statements about your decision.

	Strongly disagree	Somewhat disagree	Neither agree nor disagree	Somewhat agree	Strongly agree
1. It was the right decision.	O	O	O	O	O
2. I regret the choice that was made.	O	O	O	O	O

	Strongly disagree	Somewhat disagree	Neither agree nor disagree	Somewhat agree	Strongly agree
3. I would go for the same choice if I had to do it over again.	O	O	O	O	O
4. The choice did me a lot of harm.	O	O	O	O	O
5. The decision was a wise one.	O	O	O	O	O

6. On a scale from 1 to 5, where 1 means "not at all affordable" and 5 means "very affordable," how affordable did you think the cost of genetic testing was?

Not at all affordable		Somewhat affordable		Very Affordable
1	2	3	4	5

7. Did you have any concerns about the billing process?
   O Yes
   O No
   O Don't know

If yes, what concerns? ____
____

8. Did you have any concerns about the insurance process?
   O Yes
   O No
   O Don't know

If yes, what concerns? ____
____

9. On a scale from 1 to 5, where 1 means "not at all" and 5 means "very much" how much did the coronavirus (COVID-19) affect your decision to receive genetic testing?

Not at all		Somewhat		Very much
1	2	3	4	5

Section 5:

Next, we would like to ask you your thoughts about how likely you are to develop different types of cancer.

1. Compared to other people your gender, age, and race, how likely do you think you are to get each of the following types of cancer in your lifetime?

	A lot less likely	Somewhat less likely	About as likely	Somewhat more likely	A lot more likely
a. Breast cancer	O	O	O	O	O
b. Ovarian cancer [if female]	O	O	O	O	O
c. Colon cancer	O	O	O	O	O

2. On a scale of 0-100%, what do you believe is your chance of getting breast cancer sometime in your lifetime?

   [ ][ ][ ] %

3. [If female] On a scale of 0-100%, what do you believe is your chance of getting ovarian cancer sometime in your lifetime?

   [ ][ ][ ] %

4. On a scale of 0-100%, what do you believe is your chance of getting colon cancer sometime in your lifetime?

   [ ][ ][ ] %

5. What recommendations did you receive from a genetic counselor to reduce your risk of cancer? Select all that apply.
   a. Choose an item.

6. How did you initially receive these recommendations from the genetic counselor?
   O By phone
   O In person
   O By mail
   O Through MyChart
   O Other ________________________________

Section 6:
We would next like to ask some questions about who you might have talked to about your genetic test results <u>in the last 4 weeks.</u>

1. Have you talked with your primary care provider about your genetic test results?
   - O Yes
   - O No
   - O Don't know

If yes, did your primary care provider make any recommendations to reduce your risk of cancer?
   - O Yes
   - O No
   - O Don't know

If yes, what recommendations? Select all that apply.
   a. Choose an item.

2. Have you talked with any other health care providers about your genetic test results in the last 4 weeks?
   - O Yes
   - O No
   - O Don't know

If yes, what type of health care provider? ____

3. Have you talked with your family members about your genetic test results?
   - O Yes
   - O No
   - O Don't know

If yes, with which family members did you discuss this information?

O Mother	O Aunts	O Nieces
O Father	O Uncles	O Nephews
O Sisters	O Sons	O Cousins
O Brothers	O Daughters	O Other _______

4. Have you talked with anyone else about your genetic test results?
   - O Yes
   - O No
   - O Don't know

If yes, who did you talk to? ____

Section 7:

For the next set of questions, we'd like to find out more about how patients think about inherited risk. Please mark whether you agree or disagree with the following statements.

	Agree	Disagree	Don't know
1. Knowing about inherited risk (passed down within a family) can affect choices about cancer treatments (for example, medications or surgery).	O	O	O
2. People with an inherited risk for cancer (and their at-risk relatives) are more likely to develop more than one type of cancer.	O	O	O

	Agree	Disagree	Don’t know
3. A person with inherited risk for cancer will definitely get cancer one day.	O	O	O
4. The lifetime chance of getting cancer depends on which altered cancer gene is inherited.	O	O	O
5. People with an inherited risk for cancer may get cancer at a younger age than people with average risk.	O	O	O
6. In the future, more information could become available that could alter the meaning of genetic test results.	O	O	O
7. Female-specific cancer risk, such as ovarian cancer, can generally be passed on from either the father or mother.	O	O	O
8. The blood relatives (for example, sister, father, or child) of a person with a mutation in a cancer risk gene might share the same gene mutation.	O	O	O
9. A person with an inherited risk for cancer may have distant relatives (for example, cousins) who also have increased cancer risk.	O	O	O
10. All children of a person with inherited cancer risk will also have inherited cancer risk.	O	O	O
11. In most cases, the sisters and brothers of a person with inherited cancer risk have a 50-50 (50%) chance of having inherited risk for cancer too.	O	O	O
12. All of the gene mutations that could increase risk for cancer have been discovered.	O	O	O
13. If a person does not have a mutation found on genetic testing (negative result), interpreting results will depend on whether someone in the family has a known gene mutation associated with cancer risk (positive result).	O	O	O
14. Some gene mutations mean a larger increase in the risk for cancer while others mean a smaller increase in the risk for cancer.	O	O	O
15. A Variant of Uncertain Significance (VUS) will not likely influence recommendations for screening or prevention.	O	O	O
16. Multi-gene panel testing could find a mutation in a gene that is not clearly associated with the pattern of cancer in the family.	O	O	O

Section 8:

For the next set of questions, we'd like to ask more about you.

1. What is the highest level of school that you have completed?
   - O Elementary school
   - O Junior high or some High school
   - O Some college of Associate degree
   - O College degree
   - O Graduate degree

2. What is your ethnicity?
   - O Latino/Hispanic
   - O Non-Hispanic/non-Latino

3. What is your race? (Check all that apply)
   - O White/Caucasian
   - O African-American/Black
   - O Asian/Pacific Islander
   - O Native American/Alaska Native
   - O Other __________________________

4. Do your parents or grandparents have any Ashkenazi (Eastern European) Jewish ancestry?
   - O Yes
   - O No
   - O Not sure

5. What is your marital status?
   - O Married
   - O Living as married
   - O Widowed
   - O Divorced
   - O Separated
   - O Never been married

6. What is your zip code? ________

7. Think about your household's total income. About how much did your household receive in the last year?
   - O Less than $25,000
   - O $25,000 - $49,999
   - O $50,000 - $74,999
   - O $75,000 - $99,999
   - O $100,000 or higher

8. Do you currently have health insurance?
   - O Yes, I have private insurance (for example, through my job, HMO)
   - O Yes, I have public insurance (for example, Medicaid, Medicare)
   - O No

9. Do you have a doctor, nurse, or other practitioner who you think of as your primary care provider?
   - O Yes
   - O No
   - O Not sure

On a scale from 1 to 7, where 1 means "not true of me at all" and 7 means "very true of me," how true do you feel the following statements are of you:

	1 Not true of me at all	2	3	4	5	6	7 Very true of me
10. The Coronavirus (COVID-19) has impacted me negatively from a financial point of view.	O	O	O	O	O	O	O
11. I have lost job-related income to the Coronavirus (COVID-19).	O	O	O	O	O	O	O
12. I have become depressed because of the Coronavirus (COVID-19).	O	O	O	O	O	O	O
13. The Coronavirus (COVID-19) outbreak has impacted my psychological health negatively.	O	O	O	O	O	O	O
14. Thinking about the coronavirus (COVID-19) makes me feel threatened.	O	O	O	O	O	O	O
15. I am afraid of the coronavirus (COVID-19).	O	O	O	O	O	O	O
16. I am stressed around other people because I worry I'll catch the coronavirus (COVID-19).	O	O	O	O	O	O	O

17. Do you think you have had the Coronavirus (COVID-19)?
O No
O Yes
O Maybe

18. Has a healthcare provider ever told you that you have the Coronavirus (COVID-19)?
O Yes, definitely
O Yes, probably or suspected
O No

19. Has anyone else in your household been told by a healthcare provider that they have the Coronavirus (COVID-19)?
O Yes, definitely
O Yes, probably or suspected
O No

Section 9:

Finally, we are interested in how patients prefer to learn information. For the last set of questions, please circle the number that best reflects how good you are at doing the following things:

1. How good are you at working with fractions?

Not at all Good					Extremely Good
1	2	3	4	5	6

2. How good are you at working with percentages?

Not at all Good					Extremely Good
1	2	3	4	5	6

3. How good are you at calculating a 15% tip?

Not at all Good					Extremely Good
1	2	3	4	5	6

4. How good are you at figuring out how much a shirt will cost if it is 25% off?

Not at all Good					Extremely Good
1	2	3	4	5	6

5. When reading the newspaper, how helpful do you find tables and graphs that are parts of a story? (6-point Likert-type scale: not at all helpful – extremely helpful)

Not at all Helpful					Extremely Helpful
1	2	3	4	5	6

6. When people tell you the chance of something happening, do you prefer that they use words ("it rarely happens") or numbers ("there's a 1% chance")? (6-point Likert-type scale: always prefer words – always prefer numbers)

Always Prefer Words					Always Prefer Numbers
1	2	3	4	5	6

7. When you hear a weather forecast, do you prefer predictions using percentages (e.g., "there will be a 20% chance of rain today") or predictions using only words (e.g., "there is a small chance of rain today")? (6-point Likert-type scale: always prefer words – always prefer numbers)

Always Prefer Words					Always Prefer Numbers
1	2	3	4	5	6

8. How often do you find numerical information to be useful? (6-point Likert-type scale: never -always)

Never					Always
1	2	3	4	5	6

Section 10:

That is the end of the questions. Do you have anything else that you would like to share with the research team?

Thank you again for agreeing to share this information with us.
