# Supplementary material for: Comparing models of delivery for cancer genetics services among patients receiving primary care who meet criteria for genetic evaluation in two healthcare systems: BRIDGE randomized controlled trial
Source: BMC Health Serv Res. 2021 Jun 2;21:542. doi: 10.1186/s12913-021-06489-y (PMC8170651; doi:10.1186/s12913-021-06489-y)
Supplement: Supplementary file 1 — Additional file 1. [file 12913_2021_6489_MOESM1_ESM.zip › 12913_2021_6489_MOESM1_ESM/Supplemental File Questionnaire 2 _ESM.pdf]

Questionnaire #2 – Participants who receive genetic testing

Our team is doing a study called BRIDGE to better understand how to deliver genetics care to patients and we are interested in hearing your thoughts. You will find additional information about the study attached to the invitation email. If you would like to participate in the study, please complete this questionnaire.

For the first set of questions, we would like you to think about the genetic test results that you received.

Section 1:

1. What was your genetic test result?
   - O Positive
     What would you say is the meaning of the positive result?
     - O A genetic mutation was identified and, therefore, I will develop cancer.
     - O A genetic mutation was found and, therefore, I have an increased chance of developing cancer.
   - O Negative
     What would you say is the meaning of the negative result?
     - O No genetic mutation was identified and, therefore, I will not develop cancer.
     - O No genetic mutation was found and, therefore, I am less likely to develop cancer.
     - O No genetic mutation was identified, and these results give me no information about my risk for developing cancer.
   - O VUS
     What would you say is the meaning of the VUS result?
     - O A genetic change was found but it is not certain what effect this has on my chance of developing cancer.
     - O A genetic change was found that means that I have an increased chance of developing cancer.
     - O A genetic change was found that means that I have a decreased chance of developing cancer.
   - O My test results are not clear to me.

2. Did your genetic test results indicate that you were at increased risk of:

Breast cancer?	YES	NO	DON'T KNOW
Ovarian cancer? (If female)	YES	NO	DON'T KNOW
Colon cancer?	YES	NO	DON'T KNOW
Any other cancer?	YES	NO	DON'T KNOW

<u>If yes to any other cancer</u>, which cancer(s)? Select all that apply.
   a. Choose an item.

3. On a scale from 1 to 5, where 1 means "not at all" and 5 means "all of the time," how often would you say you have thought about your test results since you received them?

Not at all		Some of the time		All of the Time
1	2	3	4	5

Section 2:

Next, we would like to ask you your thoughts about how likely you are to develop different types of cancer.

1. Compared to other people your gender, age, and race, how likely do you think you are to get each of the following types of cancer in your lifetime?

	A lot less likely	Somewhat less likely	About as likely	Somewhat more likely	A lot more likely
a. Breast cancer	O	O	O	O	O
b. Ovarian cancer [if female]	O	O	O	O	O
c. Colon cancer	O	O	O	O	O

2. On a scale of 0-100%, what do you believe is your chance of getting breast cancer sometime in your lifetime?

			%

3. [If female] On a scale of 0-100%, what do you believe is your chance of getting ovarian cancer sometime in your lifetime?

			%

4. On a scale of 0-100%, what do you believe is your chance of getting colon cancer sometime in your lifetime?

			%

Section 3:

For the next set of questions, we would like to ask who you might have talked to about your genetic test results.

1. Have you talked with your primary care provider about your genetic test results in the past year?
   O Yes
   O No
   O Don't know

   If yes, did your primary care provider make any recommendations to reduce your risk of cancer?
   O Yes
   O No
   O Don't know

If yes, what recommendations? Select all that apply.

a. Choose an item.

2. Have you talked with any other health care providers about your genetic test results in the past year?

O Yes
O No
O Don't know

If yes, what type of health care provider? ____

3. Have you talked with your family members about your genetic test results?

O Yes
O No
O Don't know

If yes, with which family members did you discuss this information? Select all that apply.

O Mother	O Aunts	O Nieces
O Father	O Uncles	O Nephews
O Sisters	O Sons	O Cousins
O Brothers	O Daughters	O Other

4. If you shared your genetic test results with your family members, what were their initial responses to your results? Select all that apply.

O Sad
O Happy
O Relieved
O Anxious
O Concerned
O Confused
O Ambivalent
O Angry
O Other ____

5. Have you talked with anyone else about your genetic test results in the last 12 months?

O Yes
O No
O Don't know

If yes, who did you talk to? ____

Section 4:

There are number of different things that individuals might do to lower their risk of developing cancer. In the next set of questions please mark if you have had any of the following screening tests <u>in the last year because of your genetic test results.</u>

	Yes	No	Don't know
1. A mammogram, meaning an x-ray of your breasts to look for breast cancer?	O	O	O
2. A breast MRI?	O	O	O

	Yes	No	Don't know
3. A breast ultrasound?	○	○	○
4. A clinical exam of your breasts by a healthcare provider?	○	○	○
5. A breast self-exam, meaning you examined your breasts yourself?	○	○	○
6. A colonoscopy?	○	○	○
7. Upper endoscopy?	○	○	○
8. A pancreatic screening by endoscopic ultra sound (EUS)?	○	○	○
9. A pancreatic screening by cholangiopancreatography (ERCP)?	○	○	○
10. A PSA (prostate-specific antigen) test?	○	○	○
11. A FIT or FOBT test (also called a stool card or Cologuard)?	○	○	○
12. A pelvic ultrasounds (uterus, ovaries, or other gynecological organs?	○	○	○
13. A blood test called CA-125?	○	○	○
14. Other? ________________________________	○	○	○

15. On a scale from 1 to 5, where 1 means "not at all" and 5 means "very much" to what extent did the coronavirus (COVID-19) affect whether you were able to have the screening tests listed above?

Not at all		Somewhat		Very much
1	2	3	4	5

16. Have you had a surgical procedure to reduce your risk for cancer?

O YES O NO O DON'T KNOW

If yes, what surgical procedure(s) did you have done?

a. Choose an item.

In addition to screening, there are other things individuals might do to lower their risk of developing cancer. In the next set of questions, please indicate if you have done any of the following in the last year because of your genetic test results.

	Yes	No	Don't know
17. Received a medication to prevent cancer, such as Tamoxifen or Raloxifene?	O	O	O
18. Started taking any other new medications thought to reduce cancer risk? If yes, what?	O	O	O
19. Started taking multivitamins?	O	O	O
20. Started taking new natural products thought to reduce cancer risk (i.e., supplements or other natural therapies)? If yes, what?	O	O	O
21. Changed your alcohol consumption?	O	O	O
22. Attempted to quit smoking?	O	O	O
23. Quit smoking?	O	O	O
24. Changed your eating habits?	O	O	O
25. Changed your exercise habits?	O	O	O
26. Attempted to achieve or maintained a healthy body weight?	O	O	O
27. Any other changes to reduce risk of cancer? ______________________________	O	O	O

28. Have you been diagnosed with cancer in the past year?

O YES　　　O NO　　　O DON'T KNOW

If yes, what were you diagnosed with? Select all that apply.

a. Choose an item.

29. Have you had other genetic testing in the last year?

O YES　　　O NO　　　O DON'T KNOW

30. Have you experienced discrimination in any of the following ways following genetic testing? (Select all that apply)
    - O People changing the way they feel about me and interact with me
    - O Problems with life insurance
    - O Problems with long-term care insurance
    - O Problems with disability insurance
    - O Problems with work
    - O Other ____
    - O None of these

Section 5:

For the last set of questions, we'd like to ask more about how you might search for and think about health information.

	Strongly disagree	Somewhat disagree	Neither agree nor disagree	Somewhat agree	Strongly agree
1. I know **what** health resources are available on the Internet.	O	O	O	O	O
2. I know **where** to find helpful health resources on the Internet.	O	O	O	O	O
3. I know **how** to find helpful health resources on the Internet.	O	O	O	O	O
4. I know **how to use** the Internet to answer my questions about health.	O	O	O	O	O
5. I know how to use the health information I find on the Internet to help me.	O	O	O	O	O
6. I have the skills I need to evaluate the health resources I find on the Internet.	O	O	O	O	O
7. I can tell high quality health resources from low quality health resources on the Internet.	O	O	O	O	O

	Strongly disagree	Somewhat disagree	Neither agree nor disagree	Somewhat agree	Strongly agree
8. I feel confident in using information from the Internet to make health decisions.	O	O	O	O	O

9. Some newspapers or general magazines publish a special section that focuses on health. In the past 12 months, about how often have you read such health sections?
   - O Every day
   - O Several days per week
   - O 2 or 3 times per month
   - O About once per month
   - O 5 to 10 times per year
   - O Less than 5 times per year

10. Some local television news programs include special segments of their newscasts that focus on health issues. In the past 12 months, how often have you watched health segments on local news?
   - O Every day
   - O Several days per week
   - O 2 or 3 times per month
   - O About once per month
   - O 5 to 10 times per year
   - O Less than 5 times per year

11. Some people notice information about health on the Internet, even when they are not trying to find out about a health concern they have or someone in the family has. About how often have you read this sort of health information in the past 12 months?
   - O Every day
   - O Several days per week
   - O 2 or 3 times per month
   - O About once per month
   - O 5 to 10 times per year
   - O Less than 5 times per year

12. How often do you talk to friends or family members about health?
   - O Very often
   - O Somewhat often
   - O Not very often
   - O Not at all

13. In the past 30 days, how often would you say you have looked for information about ways to stay healthy or to feel better?
   - O Very often
   - O Somewhat often
   - O Not very often
   - O Not at all

On a scale from 1 to 5 where 1 is strongly disagree, and 5 is strongly agree, how much do you agree or disagree with the following statements:

	Strongly disagree	Somewhat disagree	Neither agree nor disagree	Somewhat agree	Strongly agree
14. Living life in best possible health is very important to me	O	O	O	O	O
15. Eating right, exercising, and taking preventive measures will keep me healthy for life	O	O	O	O	O
16. My health depends on how well I take care of myself	O	O	O	O	O
17. I actively try to prevent disease and illness	O	O	O	O	O
18. I do everything I can to stay healthy	O	O	O	O	O
19. I make a point to read and watch stories about health	O	O	O	O	O
20. I really enjoy learning about health issues	O	O	O	O	O
21. To be and stay healthy it's critical to be informed about health issues	O	O	O	O	O
22. The amount of health information available today makes it easier for me to take care of my health	O	O	O	O	O
23. When I take medicine, I try to get as much information as possible about its benefits and side effects	O	O	O	O	O
24. I need to know about health issues so I can keep myself and my family healthy	O	O	O	O	O
25. Before making a decision about my health, I find out everything I can about this issue	O	O	O	O	O

	Strongly disagree	Somewhat disagree	Neither agree nor disagree	Somewhat agree	Strongly agree
26. It's important to me to be informed about health issues	O	O	O	O	O
27. I know how to assess the role of genes for health.	O	O	O	O	O
28. I know how to assess my genetic risk for disease.	O	O	O	O	O
29. I can explain genetic issues to people.	O	O	O	O	O

30. How often do you have someone (like a family member, friend, hospital/clinic worker or caregiver) help you read hospital materials?

None of the time	A little of the time	Some of the time	Most of the time	All of the time
O	O	O	O	O

31. How often do you have problems learning about your medical condition because of difficulty understanding written information?

None of the time	A little of the time	Some of the time	Most of the time	All of the time
O	O	O	O	O

32. How confident are you filling out medical forms by yourself?

Not at all	A little bit	Somewhat	Quite a bit	Extremely
O	O	O	O	O

On a scale from 1 to 7, where 1 means "not true of me at all" and 7 means "very true of me," how true do you feel the following statements are of you:

	1 Not true of me at all	2	3	4	5	6	7 Very true of me
33. The Coronavirus (COVID-19) has impacted me negatively from a financial point of view.	O	O	O	O	O	O	O

	1 Not true of me at all	2	3	4	5	6	7 Very true of me
34. I have lost job-related income to the Coronavirus (COVID-19).	O	O	O	O	O	O	O
35. I have become depressed because of the Coronavirus (COVID-19).	O	O	O	O	O	O	O
36. The Coronavirus (COVID-19) outbreak has impacted my psychological health negatively.	O	O	O	O	O	O	O
37. Thinking about the coronavirus (COVID-19) makes me feel threatened.	O	O	O	O	O	O	O
38. I am afraid of the coronavirus (COVID-19).	O	O	O	O	O	O	O
39. I am stressed around other people because I worry I'll catch the coronavirus (COVID-19).	O	O	O	O	O	O	O

40. Do you think you have had the Coronavirus (COVID-19)?
O No
O Yes
O Maybe

41. Has a healthcare provider ever told you that you have the Coronavirus (COVID-19)?
O Yes, definitely
O Yes, probably or suspected
O No

42. Has anyone else in your household been told by a healthcare provider that they have the Coronavirus (COVID-19)?
O Yes, definitely
O Yes, probably or suspected
O No

That is the end of the questions. Do you have anything else that you would like to share with the research team?

____

Thank you again for agreeing to share this information with us.
